# Protective effects of *Salvia miltiorrhiza* injection against learning and memory impairments in streptozotocin-induced diabetic rats

**DOI:** 10.3892/etm.2014.1919

**Published:** 2014-08-19

**Authors:** HUABO CAI, LUYA LIAN, YU WANG, YUANYUAN YU, WEI LIU

**Affiliations:** 1 Department of ICU, Sir Run Run Shaw Hospital, School of Medicine, Zhejiang University, Hangzhou, Zhejiang 310016, P.R. China; 2 Department of Prosthetics, Stomatology Hospital, School of Medicine, Zhejiang University, Hangzhou, Zhejiang 310058, P.R. China

**Keywords:** diabetes, *Salvia miltiorrhiza*, mitogen-activated protein kinase phosphatase-1, learning and memory impairment

## Abstract

The aim of this study was to explore the protective effects of *Salvia miltiorrhiza* injection against learning and memory impairment in streptozotocin (STZ)-induced diabetic rats and the possible mechanism involved. Sprague Dawley male rats (n=30) were randomized into three groups: Diabetes, diabetes treated with *S. miltiorrhiza* injection and normal control. Diabetes was induced by an intraperitoneal injection of STZ (65 mg/kg). The *S. miltiorrhiza* injection-treated rats received an intraperitoneal injection of *S. miltiorrhiza* (5 ml/kg/day) while the rats of the other two groups were administered an intraperitoneal injection of the same volume of 0.9% saline for four weeks. After four weeks of treatment, the escape latency and search strategies in the rats were assessed by the Morris water maze test. The protein levels of mitogen-activated protein kinase phosphatase-1 (MKP-1) were also assessed by immunohistochemistry. Four weeks after the induction of diabetes, the body weight of the diabetic rats was significantly lower and the blood glucose concentration was significantly higher than that of the control rats. *S. miltiorrhiza* injection was observed to improve the blood glucose and learning ability (P<0.05). Compared with the control group, the expression of MKP-1 was significantly decreased in the hippocampal area of the diabetes group; *S. miltiorrhiza* injection-treated rats showed an increased expression compared with the diabetic rats, but the expression remained lower than that of the normal control group (P<0.05). In conclusion, *S. miltiorrhiza* injection can improve the learning and memory decline of diabetic rats. The changes in expression of MKP-1 under hyperglycemia may play a role in the protective effects of *S. miltiorrhiza* against dementia in diabetic rats.

## Introduction

The prevalence of diabetes is rising; furthermore, the prevalence of total diabetes (physician-diagnosed and undiagnosed) has been calculated to be 13.4% (11.7–15.0%), indicating that ~40% of diabetes cases remain undiagnosed (1). A previous study revealed that long-term hyperglycemia was associated with adverse effects on brain activity, neuronal structural changes and impaired long-term spatial memory (2). Diabetes can act on synaptic plasticity through mechanisms involved in metaplasticity. Such persistent inhibition of long-term potentiation and facilitation of long-term depression may lead to activity-dependent synapse weakening and contribute to cognitive impairments (3). Our previous study showed that hyperglycemia was associated with learning and memory decline in diabetic rats, possibly through a mechanism involving mitogen-activated protein kinase (MAPK) signaling pathways (4). The MAPK pathway is necessary for the maintenance of synaptic plasticity in the dentate gyrus, and MAPK/extracellular signal-regulated kinase (ERK) activation is required for long-term potentiation (LTP)-dependent transcriptional regulation (5).

*Salvia miltiorrhiza* is a Traditional Chinese Medicine that is widely used throughout clinics in Eastern Asia to treat liver fibrosis (6), diabetes (7), diabetic nephropathy (8), stroke and Alzheimer’s disease (AD) (9). Biochemical and pharmacological investigations have identified the phenolic acids of *S. miltiorrhiza* as the effective constituents responsible for its beneficial pharmacological activities (10). It has been proposed that *S. miltiorrhiza* may be able to improve the condition of patients with neurological disease, although the definite mechanisms remain unknown (11–13). The aim of the present study was to investigate the protective effects of *S. miltiorrhiza* injection in experimental diabetic rats, and to explore the association between MAPK phosphatase-1 (MKP-1) levels in the hippocampus and the protective effects of *S. miltiorrhiza* injection in the learning and memory ability of diabetic rats.

## Materials and methods

### Animal groups and induction of diabetes

A total of 30 male Sprague Dawley rats (Experimental Animal Center of Zhejiang University, Hangzhou, China) were used in the experiments and were randomly divided into three groups (n=10): Diabetes, *S. miltiorrhiza*-treated and normal control. The rats of the diabetes and *S. miltiorrhiza*-treated groups received a single dose of 65 mg/kg streptozotocin (STZ) (Alexis Corporation, Lausen, Switzerland) intraperitoneally (i.p.) to induce diabetes while the remaining 10 rats in the control group received an injection of an equivalent volume of 0.9% saline. Forty-eight hours after STZ injection, blood glucose levels >16.7 mmol/l and a positive urine glucose test were regarded to indicate the successful induction of diabetes. The *S. miltiorrhiza-*treated rats then received 5 ml/kg/day *S. miltiorrhiza* i.p. and rats in the other two groups received the same volume of 0.9% saline i.p. for four weeks. The study was approved by the Animal Care Committee of Zhejiang University (Hangzhou, China) and conformed to the Guide for the Care and Use of Laboratory Animals.

### Behavioral testing

Four weeks after the induction of diabetes, the learning and memory ability of the rats was assessed using the Morris water maze. The procedure included place navigation, which was used to test the rats’ access to learning and memory abilities, and the length of time taken by each rat to reach the destination. The rats were trained in the morning for four days. Subsequent to place navigation, the platform was removed and all the rats were placed into the water at the same point. The number of times the rats passed through the platform in 120 sec was measured.

### MKP-1 immunohistochemistry assay

Subsequent to the Morris water maze test, the rats from each group were anesthetized and perfused intracardially with normal saline (pH 7.0). Following saline perfusion, the animals were perfused with 300 ml fixative containing 4% paraformaldehyde in 0.1 M phosphate-buffered saline (PBS; pH 7.4). The brain of each rat was then removed, fixed in the same fixative for 4 h and placed in 30% phosphate-buffered sucrose (pH 7.4) until the tissue sank. Tissue sections (10-μm) were cut on a freezing microtome through coronary planes of the brain and mounted onto 0.02% poly-L-lysine-coated slides. The tissue sections were washed in 0.1 M PBS and incubated in 1% bovine serum albumin (BSA; pH 7.0) for 30 min. The tissues were then incubated overnight at 4°C with mouse anti-rat MKP-1 monoclonal antibodies (1:100 dilution in 0.1 M PBS plus 1% BSA; Santa Cruz Biotechnology, Inc., Santa Cruz, CA, USA). Control sections were incubated in 0.1 M PBS plus 1% BSA. The sections were washed three times for 5 min in 0.1 M PBS and then incubated with a biotinylated goat anti-mouse secondary antibody (1:200 dilution; Boster Biological Technology Ltd., Wuhan, China) for 5 h at room temperature. The sections were washed three times for 5 min in 0.1 M PBS and then incubated in an avidin-horseradish peroxide solution. The sections were subsequently dehydrated through an ethanol and xylene series, prior to the application of the cover slips.

### Statistical analysis

Hippocampal slides from each rat were examined at a magnification of ×400 and analyzed with the University of Texas Health Science Center at San Antonio ImageTool 3.0 (University of Texas Medical School, San Antonio, TX, USA). The number of MKP-1-positive cells per vision field of each rat was measured. All data are presented as the mean ± standard deviation. Statistical analysis was performed using one-way analysis of variance by SPSS^®^ statistical software, version 17.0 (SPSS Inc., Chicago, IL, USA) for Windows^®^. P<0.05 was considered to indicate a statistically significant difference.

## Results

### Body weight and blood glucose concentration

**Figure 1:**
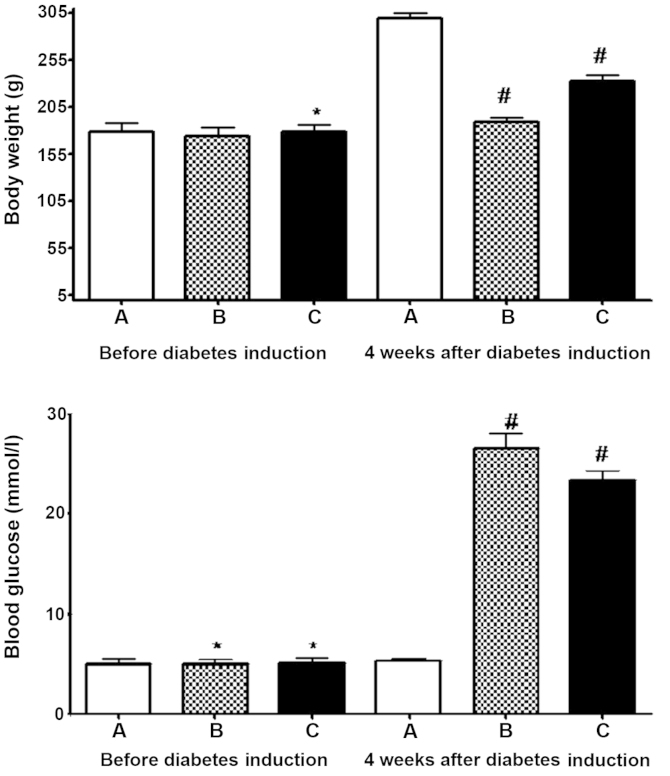
Body weight and blood sugar level of each group prior and subsequent to diabetes induction. A, normal control rats; B, diabetic rats; C, *Salvia miltiorrhiza*-treated rats. ^*^P>0.05 and ^#^P<0.05 vs. the normal rats.

Prior to the STZ injection, no significant difference was identified among the three groups with regard to body weight or blood glucose concentration. After four weeks of diabetes induction, the diabetic rats had a significantly lower body weight and significantly higher blood glucose concentration than the normal rats. The *S. miltiorrhiza* treatment was observed to increase the body weight and decrease the blood glucose concentration in the diabetic rats (P<0.01) (Fig. 1).

### Behavioral changes of the three groups four weeks after S. miltiorrhiza treatment

During the four-day training phase of the Morris water maze test, the escape latency of all rats in the platform quadrant improved gradually. The escape latency of the diabetic rats on the fourth day was markedly longer (26.3±13.2 sec) than that of the control rats (12.6±3.8 sec) (P<0.05). *S. miltiorrhiza* injection improved the escape latency of the diabetic rats to 18.6±0.1 sec (P<0.05). On the fourth day, the average platform-finding frequency in the 120-sec period was 5.6±2.3 times for the normal rats, which was higher than that for the diabetic rats (2.6±1.1 times; P<0.05). The platform-finding frequency for the *S. miltiorrhiza*-treated rats was 3.8±1.7 times (P<0.05). The longer escape latency and lower platform-finding frequency in the diabetic rats observed in the Morris water maze tests indicated that diabetes affected the learning and memory capacity and that *S. miltiorrhiza* treatment could improve the learning and memory capacity in diabetic rats.

### Assessment of MKP-1 protein in the rat hippocampus

**Figure 2:**
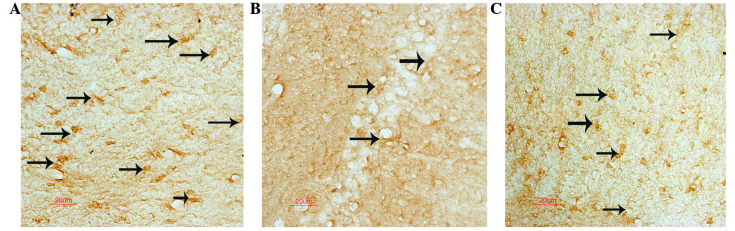
Immunohistochemical staining of MKP-1 in each group four weeks after streptozotocin induction (magnification, ×400). (A) Normal hippocampal tissue; (B) hippocampal tissue of a diabetic rat; and (C) hippocampal tissue of a *Salvia miltiorrhiza*-treated diabetic rat. The arrows show the MKP-1-positive cells with brown-stained granules. The normal hippocampal neurons exhibited higher expression levels of MKP-1 than the neurons in the diabetic rats, which showed few brown neurons. The MKP-1 expression recovered to a certain extent in the hippocampal tissue of *S. miltiorrhiza*-treated rats. MKP-1, mitogen-activated protein kinase phosphatase-1.

MKP-1-positive neurons with brown granules were observed under the microscope. The brown granules were present in the hippocampus of the normal rats, but were relatively rare in the diabetic rats, indicating that MKP-1 had a lower expression in the diabetic condition. However, the expression was enhanced in the *S. miltiorrhiza-*treated group. The number of MKP-1-positive neurons in the hippocampus of the normal rats was 22.8±4.3 cells/mm^2^, which was significantly higher than that of the diabetic rats (13.5±3.2 cells/mm^2^). *S. miltiorrhiza* treatment increased the number of MKP-1-positive neurons to 18.7±4.0 cells/mm^2^ in the diabetic rats (P<0.05) (Fig. 2).

## Discussion

Diabetic encephalopathy is one of the complications of diabetes and has been shown to differ in types 1 and 2 diabetes with regard to the nature of the resulting cognitive deficits (14). A previous study showed that a pre-existing diabetic condition doubled the incidence of dementia and AD and increased mortality. Elderly patients with diabetes developed more extensive vascular pathology, which increased dementia risk. Dementia risk was also increased in elderly patients with diabetes and AD-type pathology, particularly in apolipoprotein E-ɛ4 carriers (15). The present study explored the cognitive function of STZ-induced diabetic rats and found that the diabetic rats demonstrated a markedly longer escape latency and a lower platform-finding frequency in 120 sec than the normal rats. *S. miltiorrhiza* treatment could prevent the decline in the learning and memory capacity of the diabetic rats.

The MAPK family consists of the ERK, c-Jun N-terminal kinase and p38MAPK subfamilies. Previous studies have shown that MAPK signaling is involved in long-term synaptic plasticity and memory (16,17). MKP-1, an immediate-early gene product, is one of the dual-specificity phosphatases that may play an important role in the regulation of MAPK activity, and is highly inducible in response to extracellular stimuli, including growth factors, hydrogen peroxide, angiotensin II and hyperglycemia (18–20). A previous study suggested that inactivating MKP-1 in turn increased the MAPK pathway activity and resulted in rapid LTP decay and consequent deficits in hippocampal memory retention (5). We previously demonstrated that the MAPK signaling pathway is involved in the decline in learning and memory under hyperglycemia, which led to the conclusion that MKP-1 may be associated with the diabetes-related learning and memory deficits (4). The present study showed that MKP-1 protein expression was significantly lower in the hippocampus of diabetic rats than that in the normal rats, while the expression in the *S. miltiorrhiza*-treated rats was higher than that in the diabetes group.

*S. miltiorrhiza* can activate blood flow and eliminate stasis, improve the microcirculation and reverse the upregulation of vascular endothelial growth factor induced by high glucose concentrations, in addition to ameliorating mitochondrial oxidative stress (21–24). With regard to neuronal protection, *S. miltiorrhiza* can significantly improve spatial cognition in a rat model of AD, which may be associated with a reduction in β-amyloid precursor protein expression in the rat brain (25). It has also been found that pretreatment with *S. miltiorrhiza* protects primary rat cortical neurons against H_2_O_2_-induced cytotoxicity. Furthermore, Tanshinone IIA, a phenolic acid of *S. miltiorrhiza,* has been shown to markedly reduce the increase in Ca^2+^ levels evoked by H_2_O_2_ and reverse H_2_O_2_-induced hippocampal LTP impairment (26). Previous studies have demonstrated that the long-term use of *S. miltiorrhiza* extract can not only decrease the volume of cerebral infarction but also improve learning and memory capacity; the underlying mechanism for these effects may be associated with the antioxidant property of *S. miltiorrhiza* (27,28). In the present study, *S. miltiorrhiza*-treated (four weeks, i.p) diabetic rats showed improvements in body weight and blood sugar level and an increased learning and memory capacity, as demonstrated by reductions in escape latency and increased platform-crossing frequency in 120 sec, compared with untreated diabetic rats.

In conclusion, the present study demonstrated that hyperglycemia can cause a decline in learning and memory via the negative feedback regulation of MKP-1 towards the MAPK signaling pathway. However, treatment with *S. miltiorrhiza* injection can improve the learning and memory abilities in diabetic rats and increase MKP-1 protein expression levels under the hyperglycemic condition. It is possible that *S. miltiorrhiza* injection may prevent progressive neuronal injury in diabetic rats by increasing MKP-1 protein levels.

## References

[1] Leong A, Dasgupta K, Chiasson JL, Rahme E (2013). Estimating the population prevalence of diagnosed and undiagnosed diabetes. Diabetes Care.

[2] Malone JI, Hanna S, Saporta S (2008). Hyperglycemia not hypoglycemia alters neuronal dendrites and impairs spatial memory. Pediatr Diabetes.

[3] Artola A (2008). Diabetes-, stress- and ageing-related changes in synaptic plasticity in hippocampus and neocortex - the same metaplastic process?. Eur J Pharmacol.

[4] Zhou J, Wang L, Ling S, Zhang X (2007). Expression changes of growth-associated protein-43 (GAP-43) and mitogen-activated protein kinase phosphatase-1 (MKP-1) and in hippocampus of streptozotocin-induced diabetic cognitive impairment rats. Exp Neurol.

[5] Davis S, Vanhoutte P, Pages C, Caboche J, Laroche S (2000). The MAPK/ERK cascade targets both Elk-1 and cAMP response element-binding protein to control long-term potentiation-dependent gene expression in the dentate gyrus in vivo. J Neurosci.

[6] Liu L, Wei J, Huo X (2012). The *Salvia miltiorrhiza* monomer IH764-3 induces apoptosis of hepatic stellate cells *in vivo* in a bile duct ligation-induced model of liver fibrosis. Mol Med Rep.

[7] Huang M, Xie Y, Chen L (2012). Antidiabetic effect of the total polyphenolic acids fraction from *Salvia miltiorrhiza* Bunge in diabetic rats. Phytother Res.

[8] Lee SH, Kim YS, Lee SJ, Lee BC (2011). The protective effect of *Salvia miltiorrhiza* in an animal model of early experimentally induced diabetic nephropathy. J Ethnopharmacol.

[9] Yu XY, Lin SG, Chen X (2007). Transport of cryptotanshinone, a major active triterpenoid in *Salvia miltiorrhiza* Bunge widely used in the treatment of stroke and Alzheimer’s disease, across the blood-brain barrier. Curr Drug Metab.

[10] Yuan Y, Liu Y, Lu D (2009). Genetic stability, active constituent, and pharmacoactivity of *Salvia miltiorrhiza* hairy roots and wild plant. Z Naturforsch C.

[11] Kim DH, Park SJ, Kim JM (2011). Cognitive dysfunctions induced by a cholinergic blockade and Aβ 25–35 peptide are attenuated by salvianolic acid B. Neuropharmacology.

[12] Zheng CS, Xu XJ, Ye HZ (2013). Computational pharmacological comparison of *Salvia miltiorrhiza* and *Panax notoginseng* used in the therapy of cardiovascular diseases. Exp Ther Med.

[13] Yu XY, Lin SG, Zhou ZW (2007). Tanshinone IIB, a primary active constituent from *Salvia miltiorrhiza*, exhibits neuro-protective activity in experimentally stroked rats. Neurosci Lett.

[14] Sima AA (2010). Encephalopathies: the emerging diabetic complications. Acta Diabetol.

[15] Ahtiluoto S, Polvikoski T, Peltonen M (2010). Diabetes, Alzheimer disease, and vascular dementia: a population-based neuropathologic study. Neurology.

[16] Thomas GM, Huganir RL (2004). MAPK cascade signalling and synaptic plasticity. Nat Rev Neurosci.

[17] Kelleher RJ, Govindarajan A, Jung HY, Kang H, Tonegawa S (2004). Translational control by MAPK signaling in long-term synaptic plasticity and memory. Cell.

[18] Racz B, Hanto K, Tapodi A (2010). Regulation of MKP-1 expression and MAPK activation by PARP-1 in oxidative stress: a new mechanism for the cytoplasmic effect of PARP-1 activation. Free Radic Biol Med.

[19] Calò LA, Schiavo S, Davis PA (2010). Angiotensin II signaling via type 2 receptors in a human model of vascular hyporeactivity: implications for hypertension. J Hypertens.

[20] Takehara N, Kawabe J, Aizawa Y, Hasebe N, Kikuchi K (2000). High glucose attenuates insulin-induced mitogen-activated protein kinase phosphatase-1 (MKP-1) expression in vascular smooth muscle cells. Biochim Biophys Acta.

[21] Han B, Zhang X, Zhang Q (2011). Protective effects of salvianolate on microvascular flow in a porcine model of myocardial ischaemia and reperfusion. Arch Cardiovasc Dis.

[22] Yang XY, Qiang GF, Zhang L (2011). Salvianolic acid A protects against vascular endothelial dysfunction in high-fat diet fed and streptozotocin-induced diabetic rats. J Asian Nat Prod Res.

[23] Qian S, Huo D, Wang S, Qian Q (2011). Inhibition of glucose-induced vascular endothelial growth factor expression by *Salvia miltiorrhiza* hydrophilic extract in human microvascular endothelial cells: evidence for mitochondrial oxidative stress. J Ethnopharmacol.

[24] Zhang X, He D, Xu L, Ling S (2012). Protective effect of tanshinone IIA on rat kidneys during hypothermic preservation. Mol Med Rep.

[25] Qin RA, Yao XX, Huang ZY (2012). Effects of compound danshen tablets on spatial cognition and expression of brain beta-amyloid precursor protein in a rat model of Alzheimer’s disease. J Tradit Chin Med.

[26] Wang W, Zheng LL, Wang F (2011). Tanshinone IIA attenuates neuronal damage and the impairment of long-term potentiation induced by hydrogen peroxide. J Ethnopharmacol.

[27] Lin LL, Wang W, Cheng MH, Liu AJ (2012). Protection of different components of Danshen in cerebral infarction in mice. CNS Neurosci Ther.

[28] Chong Y, Wang T, Wang W (2012). Down-regulation of P-glycoprotein expression contributes to an increase in Danshensu accumulation in the cerebral ischemia/reperfusion brain. Mol Med Rep.

